# Diagnostic Accuracy of the Five-Word Test for Mild Cognitive Impairment Due to Alzheimer’s Disease

**DOI:** 10.3390/neurolint14020029

**Published:** 2022-04-06

**Authors:** Chiara Fornari, Francesco Mori, Nicola Zoppi, Ilenia Libri, Chiara Silvestri, Maura Cosseddu, Rosanna Turrone, Matteo Maffi, Salvatore Caratozzolo, Barbara Borroni, Alessandro Padovani, Alberto Benussi

**Affiliations:** 1 Centre for Mind/Brain Sciences CIMeC, University of Trento, 38123 Rovereto, Italy; chiara.fornari96@gmail.com; 2 Neurology Unit, Department of Neurological and Vision Sciences, ASST Spedali Civili di Brescia, 25123 Brescia, Italy; tacio1999@gmail.com (F.M.); maura.cosseddu@gmail.com (M.C.); rosanna.turrone@gmail.com (R.T.); matteo.maffi2104@gmail.com (M.M.); salvatore.caratozzolo@hotmail.com (S.C.); bborroni@inwind.it (B.B.); alessandro.padovani@unibs.it (A.P.); 3 Neurology Unit, Department of Clinical and Experimental Sciences, University of Brescia, 25123 Brescia, Italy; n.zoppi001@unibs.it (N.Z.); i.libri@unibs.it (I.L.); c.silvestri003@unibs.it (C.S.)

**Keywords:** AD-MCI, CSF biomarkers, t-Tau, p-Tau_181_, Aβ_1–42_, amyloid-PET imaging, five-word test, mini-mental state examination

## Abstract

New diagnostic methods have been developed for the early diagnosis of Alzheimer’s disease (AD) with the primary purpose of intercepting the transition-phase (mild cognitive impairment, MCI) between normal aging and dementia. We aimed to explore whether the five-word test (FWT) and the mini-mental state examination (MMSE) are predictive for the early diagnosis of MCI due to AD (AD-MCI). We computed ROC analyses to evaluate the sensitivity and specificity of MMSE and FWT in predicting abnormal CSF (t-Tau, p-Tau_181_, Aβ_1–42_) and amyloid-PET biomarkers. AD-MCI patients showed lower MMSE and FWT scores (all *p* < 0.001) than non-AD-MCI. The best predictor of amyloid plaques’ presence at amyloid-PET imaging was the encoding sub-score of the FWT (AUC = 0.84). Both FWT and MMSE had low/moderate accuracy for the detection of pathological CSF Aβ_42_, t-Tau and p-Tau_181_ values, with higher accuracy for the t-Tau/Aβ_1–42_ ratio. In conclusion, the FWT, as a single-domain cognitive screening test, seems to be prompt and moderately accurate tool for the identification of an underlying AD neuropathological process in patients with MCI, supporting the importance of associating biomarkers evaluation in the work-up of patients with dementing neurodegenerative disorders.

## 1. Introduction

Alzheimer’s disease (AD) represents one of the most common causes of dementia [1,2,3], with cases estimated to reach 150 million worldwide in 2050 [4], due to the constant increase of elderly people as younger age mortality declines [5]. The natural history of AD encompasses a long preclinical phase, an early clinical phase (i.e., mild cognitive impairment, MCI) and a dementia phase [6]. Hence, in the last 10 years a great body of research highlighted the importance of an early AD diagnosis and of the transition phase between physiological aging and MCI [2,7]. Patients with MCI due to Alzheimer’s disease (AD-MCI) show detectable neuropathological changes due to AD and subtle cognitive deficits, without impact on daily life activities [1]. The prevalence of people over 65 years old with MCI ranges from 10% to 20% [8], and about 15% of those with MCI develop dementia after two years [9], while about 32% of patients with MCI develop dementia within five years [10]. Accordingly, the timing of an accurate diagnosis has a crucial role for the execution of preventive and therapeutic interventions [11], particularly in view of possible disease-modifying therapies [12].

For more than 20 years, AD remained a probabilistic clinical pathological syndrome [13], but with the gradual availability of biomarkers (initially non specific MRI measures of brain atrophy and PET measures of glucose hypometabolism; later CSF and PET measures of amyloid β and pathological tau [14]), new criteria were developed by the International Working Group (IWG), which incorporated biomarkers into the diagnostic assessment [7,15]. Continued evolution of these criteria resulted in the term preclinical dementia [16,17], which was intended to identify subjects with no symptoms but with positive biomarkers of amyloid β and tau pathology, with the objective of identifying those in the earliest stages who could benefit from potential disease-modifying therapies [18]. In 2018, the National Institute on Aging and Alzheimer’s Association (NIA-AA) group introduced a research framework in which subjects with positive biomarkers of amyloid β and tau were classified as having AD, regardless of the presence of symptoms [19]. Excluding clinical criteria removed the syndromic aspect of AD and its inherent non specificity. However, this shift to an entirely biological or biomarker-based entity also raised several questions and objections, particularly if applied in a clinical setting. To try and address these concerns, the IWG recently advocated a return to AD as a clinical biological entity, characterized by amyloid β and tau biomarkers plus a typical clinical phenotype [20].

Nevertheless, there is still no agreement about which cognitive screening instruments could be more sensitive and specific enough to detect AD in the early phases of disease [6]. Several neuropsychological tests using long word lists have been usually administered to differentiate the subjective memory complaints, which is a common symptom in an aging population [21], with the objective episodic memory impairment which seems to be unique of AD-MCI [22]. Indeed, the symptoms of the disease typically begin with mild memory difficulties [19], such as episodic memory loss due to the changes in the hippocampal volume [2,23], and to the disconnection of the hippocampus from the associative neocortical regions [24,25]. A previous hypothesis stated that patients with AD do not benefit from the semantic facilitation (i.e., cue) during the retrieval phase of a memory task [22,26]. Starting from this assumption, the five-word test (FWT) seems to be a valid test to assess the verbal episodic memory and the hippocampal memory trace consolidation, and it is a simple instrument for the screening of AD [24]. However, the accuracy of the FWT has not yet been assessed in patients with a biomarker supported diagnosis of AD-MCI vs. non-AD-MCI. On the other hand, the mini-mental state examination (MMSE) is widely used as a screening test in the clinical setting [11,27], for the assessment of global cognitive abilities, but its accuracy in detecting MCI is still controversial [28,29,30].

The aim of the present study was to explore whether the FWT, as a screening neuropsychological test appointed for the episodic memory assessment, may be prompt, valid, and predictive clinical marker of AD compared with the MMSE. Moreover, the ultimate purposes consist in investigating the feasibility of the screening tests administration to guide the early clinical differential diagnosis, to verify the probability of intercepting the earlier stages of the Alzheimer’s disease and, consequently, to increase the patients’ possibility of being eligible for the disease-modify therapies.

## 2. Materials and Methods

### 2.1. Patients

This study included a consecutive sample of 96 participants from the outpatient neurological Centre of Cognitive Disturbances and Dementia (CDCD) unit of the ASST Spedali Civili Hospital (Brescia, Italy), across a four-year interval (2017–2021). We enrolled all patients that reported memory complaints without functional impairment in activities daily living, that underwent FTW and MMSE testing at baseline, and that underwent amyloid-PET imaging or CSF analysis. After cognitive evaluation and biomarker assessment, patients were divided in two groups: AD-MCI and non-AD-MCI.

The study was approved by the Ethic Committee of the ASST Spedali Civili di Brescia Hospital (protocol NP4818) and was conducted in accordance with the statements of the Declaration of Helsinki.

### 2.2. Cognitive Screening Assessment

Participants underwent the cognitive assessment with two screening instruments, the five-word test (Italian validation [24]) and the mini-mental state examination [27]. MMSE scores were corrected for age and years of schooling using the Italian validation [31].

In order to carry out the FWT test, a list of 5 words in their Italian translation (strainer, lemonade, grasshopper, museum, and lorry), printed on a sheet of A4 paper, was shown to the patients, who were asked to read and, later on, to point and name out loud each item when the matching semantic category cue was verbally given by the examiner. Then, after removing the sheet the subjects were requested to recall the words; when one or more words were not spontaneously recalled, the semantic category cue was given in order to stimulate the item’s retrieval. An immediate recall score (IRS) was obtained by adding the number of spontaneously retrieved items to those that were retrieved thanks to the semantic cue. If the subjects failed again to recollect any words, the sheet would be shown and removed again until the missing items were identified and retrieved (max. 3 repetitions) to ensure the possibility to proceed with the second phase; this step had no impact on the individual IRS [32,33]. During the subsequent 5 min, subjects performed some nonverbal interference tasks (clock drawing test, copying of the pentagons as part of MMSE); then, a delayed recall was proposed to the subjects using the same procedure as before, providing a delayed recall score (DRS: number of retrieved items at delayed free + cued recall). The sum of the immediate free, immediate cued, delayed free and delayed cued recalls is called the total recall score (TRS), with a range from 0 to 10.

Furthermore, we assessed functional independence using the basic (BADL) and instrumental activities of daily living (IADL) questionnaires [34,35]. We also evaluated behavioral and psychological symptoms using the neuropsychiatric inventory questionnaire (NPI) [36].

### 2.3. Amyloid-PET Imaging

To investigate amyloid burden, PET amyloid imaging was acquired using 370 MBq (10 mCi) of 18F-florbetapir or 18F-flutemetamol, following the procedures provided by the ligand manufacturer, as previously reported [37]. Amyloid burden was expressed in presence/absence of amyloid plaques.

### 2.4. CSF Biomarkers

CSF was obtained during routine diagnostic lumbar puncture according to a standardized protocol, in the outpatient clinic, from 09:30 to 10:30, after informed written consent had been obtained. CSF was collected in sterile polypropylene tubes and gently mixed to avoid gradient effects. Routine chemical measures were determined. The remaining CSF was centrifuged for 3 min at 3000 rpm, and aliquots were stored at −80 °C or in liquid nitrogen for subsequent dosages. CSF concentrations were measured in duplicate by an ELISA test (Innotest hTau antigen kit and Innotest phospho-tau 181P; Abeta42, Innogenetics, Ghent, Belgium). Inter-assay variability was less than 7%. According to our laboratory standards, the cut-off value was defined as Aβ_1–42_ < 650 ng/L, total tau (t-Tau) > 400 ng/L and phosphorylated tau_181_ (p-Tau_181_) > 60 ng/L [38].

### 2.5. Statistical Analysis

Continuous variables were reported as median and interquartile range and were compared using the nonparametric Mann-Whitney U-test, after testing for normality using the Shapiro-Wilk test. Categorical variables were summarized through frequency and percentage and were compared using the Fisher’s Exact. Spearman rank-order correlations were used to assess associations between neurophysiological parameters and biomarker measures.

Furthermore, receiver operating characteristic (ROC) curves were computed to determinate the sensibility and specificity of the FWT and MMSE to identify the presence/absence of AD biomarkers in CSF (Aβ_1–42_, t-Tau, p-Tau_181_) or the presence/absence of amyloid plaques PET imaging. The area under the curve (AUC) was computed to determinate the accuracy of the FWT and MMSE. AUC values range from 0 (=inaccurate) to 1 (=perfectly accurate). As a rule of thumb, 0.5 < AUC ≤ 0.75 means low accuracy; 0.75 < AUC ≤ 0.85 represents moderate accuracy; 0.85 < AUC < 1.0 means high accuracy [39]. We also computed the Youden’s index (sensitivity + specificity − 1) to identify the optimal cut-off values of the cognitive tests that allows to maximize the differences between real positive and false positive [40].

Statistical significance level was set at α = 0.05, corrected for multiple comparisons using the Benjamini-Hochberg false discovery rate (FDR) [41]. Statistical analyses were performed using SPSS version 25.0.

## 3. Results

### 3.1. Demographic Characteristics and Cognitive Assessment

96 patients [median (IQR) age 73 (69–77) years] were recruited in the present study of whom 53 (55.2%) were classified as AD-MCI [median (IQR) age 74 (69–77) years; 71.7% female], and 43 (44.8%) as non-AD-MCI [median (IQR) age 72 (70–77) years; 28.3% female], according to clinical, CSF and amyloid PET results. Groups were comparable for age and years of education (all *p* > 0.05), but not for sex (*p* = 0.002), with a higher frequency of females in the AD-MCI group. Clinical, neuropsychological and biomarker measurements are reported in Table 1.

We observed a significant difference in MMSE scores between groups (*p* < 0.001), with a median (IQR) score of 23.4 (20.7–24.9) in the AD-MCI and 25.4 (23.0–26.3) in the non-AD-MCI group.

At the FWT, we observed a significant difference in the immediate recall score (IRS) [3.0 (2.0–4.0) vs. 4.0 (3.0–5.0)], in the delayed recall score (DLR) [3.0 (1.0–4.0) vs. 4.0 (3.0 - 5.0)], and in the total recall score (TRS) [3.0 (2.0–4.0) vs. 4.0 (3.0–5.0)] between AD-MCI and non-AD-MCI, respectively (all *p* < 0.001) (see Table 1).

Regarding biomarker assessment, 75.0% of patients underwent CSF analysis, 17.7% amyloid-PET imaging and 7.3% performed both. As expected, we observed significant differences in CSF biomarkers between groups (see Table 1).

### 3.2. Correlations between CSF Biomarkers and Neuropsychological Scores

A Spearman rank-order correlation was run to assess the relationship between CSF biomarkers and MMSE and FWT scores. There was a significant positive correlation between CSF Aβ_1–42_ and MMSE and FWT (IRS, DRS and TRS) scores and a negative correlation between CSF t-Tau and p-Tau_181_, and MMSE and FWT (IRS, DRS and TRS) scores (all *p* < 0.005). We observed larger correlation coefficients between CSF parameters and FWT DRS than IRS (see Table 2).

### 3.3. Comparison between Cognitive Assessment and Biomarkers (CSF and Amyloid-PET)

We also analyzed MMSE and FWT scores relatively to the presence/absence of an abnormal biomarker according to laboratory cut-offs, regardless of group (Table 3). We found that patients with abnormal amyloid-PET imaging showed lower FWT IRS scores compared to patients with normal amyloid-PET (*p* = 0.011); on the contrary, patients with abnormal CSF Aβ_1–42_ values showed significant lower values in MMSE scores, FWT DRS and FWT TRS (all *p* = 0.005). Regarding CSF t-Tau and p-Tau_181_, we observed significant differences only for MMSE scores in patients with abnormal t-Tau levels (*p* = 0.017), and differences in both tests in patients with abnormal p-Tau_181_ levels (all *p* < 0.05).

### 3.4. Classification Accuracy

**Figure 1:**
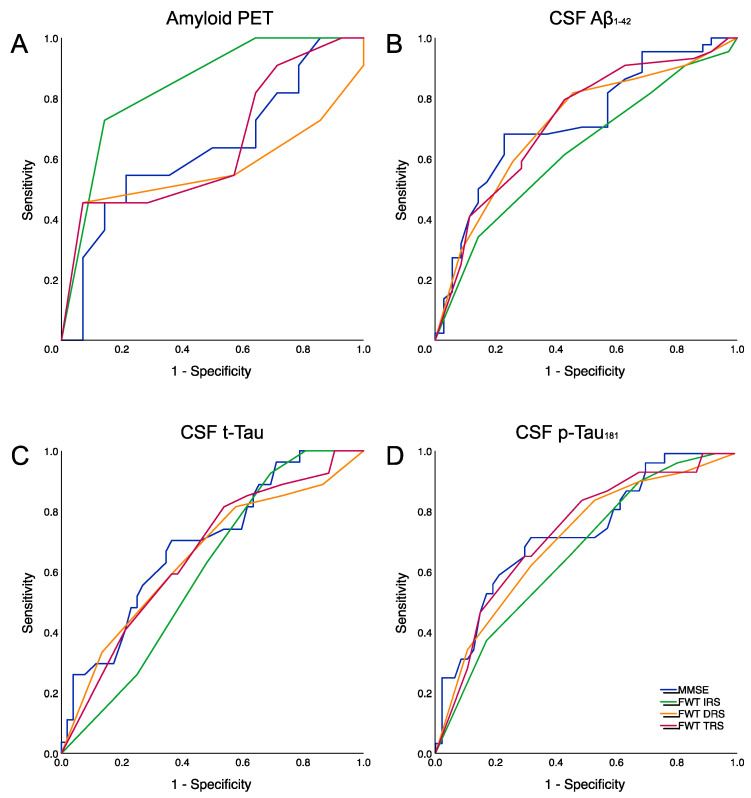
ROC curves for (**A**) amyloid-PET, (**B**) CSF Aβ_1–42_, (**C**) CSF t-Tau and (**D**) CSF p-Tau_181_. ROC: receiver operating characteristics; MMSE: mini-mental state examination (blue line); FWT: five-word test; IRS: immediate recall score (green line); DRS: delayed recall score (orange line); TRS: total recall score (purple line); CSF: cerebrospinal fluid.

In order to assess the sensitivity and specificity of MMSE and FWT for abnormal amyloid PET imaging or CSF values, we performed ROC curve analyses. We observed that the best predictor of amyloid-PET positivity was the FWT IRS [AUC 0.84 (95% CI 0.68–0.99), *p* = 0.004], while MMSE, FWT DRS and TRS performed similarly in predicting abnormal CSF Aβ_1–42_ levels, CSF p-Tau_181_ levels and, to a minor extent, also CSF t-Tau levels (see Table 4 and Figure 1). We observed higher predicting accuracy if we considered the CSF t-Tau/Aβ_1–42_ ratio > 1, with moderate classification accuracy for the FWT TRS [AUC 0.77 (95% CI 0.67–0.88), *p* < 0.001] (see Table 4 and Figure 1).

## 4. Discussion

MCI can be considered as the transition phase between normal aging and dementia [6]. The evaluation of biomarkers in the CSF or with amyloid-PET imaging are now widespread tools in clinical practice for the diagnosis of AD [20]. They allow the detection of incipient neuropathological deposition of amyloid and tau [42], to predict the risk of developing dementia and to aid in the differential diagnosis of dementing neurodegenerative disorders [43]. However, it is still unclear how to intercept the preclinical phase of disease, after the onset of neuropathological depositions. Moreover, the sensitivity and specificity of cognitive screening tests for MCI are controversial and seem to be inconsistent (e.g., MMSE [44]). Controversial results were found regarding the association between CSF biomarkers, PET amyloid [45] and clinical outcomes [46], such as memory impairment [47]. The aim of the study was to assess the validity and specificity of the FWT, a commonly used screening tests for the episodic memory assessment, compared to the MMSE for the early diagnosis of AD-MCI with a biomarker supported diagnosis. In particular, we intended to evaluate whether MMSE and FWT subscores could be accurate tools for the detection of pathological biomarkers both in the CSF (Aβ_1–42_, t-Tau and p-Tau_181_) and the presence of amyloid plaques at amyloid-PET imaging.

Aging is one of the main risk factors for the development of dementia [48,49], while the gender effect on the incidence of AD is less clear. In our study, we found gender differences in the prevalence of AD-MCI compared to non-AD-MCI. This result is coherent with previous studies that reported a higher risk of AD in women [49,50]. As expected, the AD-MCI group showed lower scores both at the MMSE and at all the sub-scores of the FWT; suggesting that AD-MCI patients present higher overall cognitive impairment and episodic memory failure in encoding [51,52] and retrieval [52] with lower sensibility to semantic cue facilitation [22,26].

Regardless of group, we found that cognitive performance measured with both MMSE and FWT correlated with CSF biomarkers, expanding the literature about the relation between FWT and biomarkers. Specifically, in accordance with a previous study in an MCI population [43], we found a positive correlation between MMSE and CSF Aβ_1–42_, and a negative one between MMSE and CSF t-Tau and p-Tau_181_. Furthermore, we found a positive correlation between immediate, delayed and total recall scores at the FWT and CSF Aβ_1–42_, and a negative one with CSF t-Tau and p-Tau_181_, suggesting that better episodic memory performance (both in encoding and retrieval phases) and higher sensibility to the semantic cue are linked to an reduced disease burden [43,47]. We also observed stronger correlations between total than immediate recall scores at the FWT and CSF biomarkers, suggesting a decreased sensitivity to cueing in patients with greater AD neuropathology.

In addition, abnormal amyloid-PET imaging was only associated with lower memory encoding ability (i.e., lower IRS FWT) [53], while no associations were found with retrieval abilities. Specifically, we found that the IRS FWT showed moderate/high accuracy for the presence of amyloid plaques at PET imaging, suggesting the close association between memory encoding impairment, amyloid plaques deposition and the lack of benefits after sematic cue facilitation. This result highlights that the FWT and, in particular, the IRS may be a sensitive clinical marker of the state-dependent neuropathological process.

Regarding CSF biomarkers, Aβ_1–42_, t-Tau and p-Tau_181_ pathological values seem to be detected with low/moderate accuracy by the MMSE and FWT, with higher accuracy for DRS and TRS sub-scores. However, considering the CSF t-Tau/Aβ_1–42_ ratio > 1, the specificity increases especially for the DRS FWT (from 42% to 64%) and TRS FWT (from 46% to 68%), in accordance with previous studies [54].

These findings provide evidence for a differential effect of abnormal amyloid deposition evaluated with CSF or amyloid PET imaging on memory scores.

We acknowledge that the present study entails some limitations. First, the number of patients that underwent CSF analysis is unbalance to those who underwent amyloid-PET imaging and only a few underwent both. Second, this is a single center study with a limited number of patients, and results should be confirmed in larger multicenter cohorts. Further research should verify these results expanding the sample and comparing the FWT scores in patients that underwent both CSF and amyloid-PET imaging analyses, and compared to a group of healthy controls.

In summary, taken together our data suggest that the discriminatory proprieties of the encoding subscore of the FWT is accurate for detecting amyloid plaque pathology at PET imaging. Moreover, MMSE and FWT scores have only moderate accuracy for detecting abnormal levels of CSF Aβ_42_, t-Tau and p-Tau_181_ values.

In conclusion, the MMSE and FWT screening tests seem to be rapid but only moderately accurate tools for the identification of an underlying AD neuropathological process, highlighting the importance of associating biomarkers evaluation in the work-up of patients with dementing neurodegenerative disorders.

## Figures and Tables

**Table 1:** Demographic and clinical characteristics of included patients according to AD-MCI and non-AD-MCI grouping.

Variables	AD-MCI (*n* = 53)	non-AD-MCI (*n* = 43)	*p* Value *
Age, years	74.0 (69.0–77.0)	72.0 (70.0–77.0)	n.s.
Sex (% female)	33 (57.9%)	13 (33.0%)	0.002
Education, years	8.0 (5.0–13.0)	8.0 (6.0–13.0)	0.023
NPI	6.0 (3.0–12.0)	10.0 (5.0–17.0)	n.s.
MMSE ^1^	23.4 (20.7–24.9)	25.4 (23.0–26.3)	<0.001
FWT (IRS)	3.0 (2.0–4.0)	4.0 (3.0–5.0)	<0.001
FWT (DLR)	3.0 (1.0–4.0)	4.0 (3.0–5.0)	<0.001
FWT (TRS)	6.0 (4.0–8.0)	8.0 (7.0–10.0)	<0.001
CSF			
Aβ_1–42_ (ng/L)	582.6 (495.8–644.5)	1135.0 (804.0–1452.0)	<0.001
t-Tau (ng/L)	677.5 (488.0–947.0)	370.0 (235.0–472.0)	<0.001
p-Tau_181_ (ng/L)	101.5 (79.5–131.0)	49.0 (38.0–67.0)	<0.001

Values are reported as median (interquartile range) or *n* (%). NPI: neuropsychiatric inventory; MMSE: mini-mental state examination; FWT: five-word test; IRS: immediate recall score; DRS: delayed recall score; TRS: total recall score; CSF: cerebrospinal fluid; n.s.: non-significant difference; ^1^ scores adjusted for age, and education level. * *p* values were calculated by Mann-Whitney U test or Fisher’s exact test, as appropriate, corrected for multiple comparisons using the Benjamini-Hochberg false discovery rate (FDR).

**Table 2:** Correlations between CSF biomarkers and neuropsychological scores.

Variables	Aβ_1–42_	t-Tau	p-Tau_181_
MMSE	*r_s_* = 0.40	*r_s_* = −0.35	*r_s_* = −0.37
	*p* < 0.001	*p* = 0.002	*p* = 0.001
FWT (IRS)	*r_s_* = 0.34	*r_s_* = −0.24	*r_s_* = −0.25
	*p* = 0.003	*p* = 0.035	*p* = 0.026
FWT (DRS)	*r_s_* = 0.37	*r_s_* = −0.39	*r_s_* = −0.41
	*p* = 0.001	*p* < 0.001	*p* < 0.001
FWT (TRS)	*r_s_* = 0.40	*r_s_* = −0.38	*r_s_* = −0.42
	*p* < 0.001	*p* = 0.001	*p* < 0.001

MMSE: mini-mental state examination; FWT: five-word test; IRS: immediate recall score; DRS: delayed recall score; TRS: total recall score; results are corrected for multiple comparisons using the Benjamini-Hochberg false discovery rate (FDR).

**Table 3:** Comparison between cognitive assessment and biomarkers (CSF and amyloid-PET).

Variables	MMSE	FWT (IRS)	FWT (DRS)	FWT (TRS)
Amyloid PET				
Abnormal	25.4 (22.3–26.4)	4.0 (2.8–4.0)	4.0 (2.0–4.0)	8.0 (5.0–9.0)
Normal	24.1 (21.7–25.3)	5.0 (4.0–5.0)	4.0 (1.0–5.0)	8.0 (7.0–10.0)
*p* value	n.s.	*p* = 0.011	n.s.	n.s.
CSF Aβ_1–42_				
Abnormal	23.2 (19.0–24.2)	3.0 (2.0–4.0)	2.0 (1.0–4.0)	6.0 (4.0–8.0)
Normal	25.1 (22.7–26.2)	4.0 (3.0–5.0)	4.0 (3.0–5.0)	8.0 (7.0–9.8)
*p* value	*p* = 0.005	n.s.	*p* = 0.005	*p* = 0.005
CSF t-Tau				
Abnormal	23.4 (21.0–25.2)	3.0 (2.0–4.8)	3.0 (1.0–4.0)	7.0 (4.0–8.0)
Normal	25.2 (22.7–26.4)	4.0 (3.0–5.0)	4.0 (3.0–5.0)	8.0 (7.0–10.0)
*p* value	*p* = 0.017	n.s.	n.s.	n.s.
CSF p-Tau_181_				
Abnormal	23.4 (20.3–24.7)	3.0 (2.0–4.0)	3.0 (1.0–4.0)	6.0 (4.0–8.0)
Normal	25.4 (22.8–26.4)	4.0 (3.0–5.0)	4.0 (3.0–5.0)	8.0 (7.0–10.0)
*p* value	*p* = 0.001	*p* = 0.010	*p* = 0.001	*p* < 0.001

MMSE: mini-mental state examination; FWT: five-word test; IRS: immediate recall score; DRS: delayed recall score; TRS: total recall score; CSF: cerebrospinal fluid; n.s.: non-significant difference; *p* values were calculated by Mann-Whitney U test corrected for multiple comparisons using the Benjamini-Hochberg false discovery rate (FDR).

**Table 4:** Classification accuracy of MMSE and FWT according to biomarkers.

Variables	AUC (95% CI)	*p* Value	Sensitivity *	Specificity *
Amyloid PET				
MMSE	0.63 (0.41–0.86)	n.s.	0.55	0.79
FWT IRS	0.84 (0.68–0.99)	0.004	0.73	0.86
FWT DRS	0.56 (0.31–0.82)	n.s.	0.46	0.93
FWT TRS	0.64 (0.42–0.87)	n.s.	0.46	0.93
CSF Aβ_1–42_				
MMSE	0.72 (0.61–0.84)	0.001	0.68	0.77
FWT IRS	0.63 (0.50–0.75)	n.s.	0.34	0.86
FWT DRS	0.71 (0.60–0.83)	0.001	0.82	0.54
FWT TRS	0.72 (0.61–0.84)	0.001	0.80	0.57
CSF t-Tau				
MMSE	0.69 (0.57–0.81)	0.005	0.70	0.64
FWT IRS	0.60 (0.48–0.73)	n.s.	0.93	0.31
FWT DRS	0.65 (0.52–0.78)	0.029	0.82	0.42
FWT TRS	0.66 (0.53–0.78)	0.021	0.82	0.46
CSF p-Tau_181_				
MMSE	0.73 (0.62–0.84)	0.001	0.72	0.68
FWT IRS	0.67 (0.55–0.79)	0.012	0.91	0.32
FWT DRS	0.71 (0.59–0.83)	0.002	0.84	0.47
FWT TRS	0.73 (0.62–0.85)	<0.001	0.66	0.70
CSF t-Tau/Aβ_1–42_ ratio > 1				
MMSE	0.68 (0.57–0.80)	0.007	0.45	0.93
FWT IRS	0.64 (0.51–0.76)	0.045	0.61	0.61
FWT DRS	0.78 (0.67–0.88)	<0.001	0.82	0.64
FWT TRS	0.77 (0.67–0.88)	<0.001	0.80	0.68

AUC: area under the curve; 95% CI: 95% confidence interval; MMSE: mini mental state examination; FWT: five-word test; IRS: immediate recall score; DRS: delayed recall score; TRS: total recall score; CSF: cerebrospinal fluid; n.s.: non-significant. * Sensitivity and specificity were computed using Youden’s index.

## Data Availability

The data presented in this study are available on request from the corresponding author. The data are not publicly available due to privacy protection.
